# 

**Published:** 1886-11-20

**Authors:** 


### CONTENTS.

**Country Physicians:** The Colchester Imbroglio - 121

**Hospital Promotion:** A Wag's Experience - 122

**Alleged Cruelty of a Hospital Sister** - 123

**The Modern Workhouse Infirmary** - 124

**The Hospitals Association.**—The Conversazione - 126

**Notes and News.**—Donation to the Longmore Hospital by the Queen.—Miscellaneous.—Brackley Cottage Hospital.—New Eye Hospital for Cardiff.—Musical Entertainments at Brompton Hospital.—The Mogg Memorial Hospital.—Llanelly Hospital.—Champagne at the Eastern Hospital.—Fever and Small-pox Hospitals.—Reopening of the West London Hospital.—Vacancies.—The Kensington District Nursing Association, etc., etc. - 128

**Annotations.**—Wanted, £50,000.—Cottage Hospitals.—Lady Doctors and Nurses.—Teeth at the General and Special Hospitals - 131

**Words of Consolation.**—VIII. Patience - 132

**Poetry.**—The Modern Samaritan - 132

**Reminiscences of the Insane.**—By a Mental Physician - 133

**The Quilter Prizes.**—Conditions - 134

**Till the Doctor Comes.**—IV. Children's Food and Management - 134

**The Ethics of the Sewage Question** - 135

**The Best Colour for Sick-room Walls** - 135

**The Editor's Letter-Box.**—Nurses' Remuneration and Pensions.—"A Few November Flowers."—Employment for Epileptics.—The Training of Midwives.—Children's Hospitals in England - 136

**The Children's Corner.**—Puzzles, Answers, etc. - 137

**The Hospital Charity Box.**—Prizes and Results - 137

**In- and Out-Patients' Hospital Diary** - 138

